# Bonding as a Positive Youth Development Construct: A Conceptual Review

**DOI:** 10.1100/2012/481471

**Published:** 2012-05-02

**Authors:** Tak Yan Lee, David P. P. Lok

**Affiliations:** Department of Applied Social Studies, College of Liberal Arts and Social Sciences, City University of Hong Kong, Hong Kong

## Abstract

The concept of bonding as a positive youth development construct is reviewed in this paper. The goals are fourfold. First, theoretical perspectives of bonding are delineated. Secondly, the relationships among bonding to caregivers, friends, romantic partners, as well as teachers, and adolescents' positive developmental outcomes are reviewed. Thirdly, with theoretical and empirical support, a discussion on how to promote bonding among adolescents is offered. Finally, a critical review on the cultural issues of bonding is provided.

## 1. Background

Extensive literature and research indicate that bonding is crucial for adolescents' healthy development. Theorists and empirical studies [1–4] suggest that social and emotional support from the family, peers, the school, and the community is important for adolescents who are in a transitional development period. In a study based on interviews of a randomized sample of 10,000 US youths across social classes [4], bonding to parents and school was identified as protective factors mitigating the numerous developmental risks faced by adolescents. Besides, bonding with healthy adults is also positively related to adolescents' psychological health and acts as a protective factor for the adolescent [5–8]. Based on an earlier version of the first author's work [9], the present paper reviews the theoretical conceptualization of bonding and its relationship with positive youth developmental outcomes. Based on theory and empirical evidence, ways of enabling adolescents to optimize bonding to significant others that are pertinent to positive development are outlined. However, the development of attachment would seem to be a necessary, universal bio-psychosocial requirement to be found in all cultures under normal circumstances as a species-specific consequence of our phylogenetic heritage [10]. However, even if the attachment system is universal, there are cultural differences since the biological system of attachment is interwoven with cultural practices [11]. Against this background, a critical discussion of cultural differences in the phenomenon of bonding is needed to illuminate the role of culture in this universal phenomenon. 

## 2. Definition of Bonding

Bonding refers to the emotional attachment and commitment an individual makes to social relationships with parents, caregivers, siblings, peers, schoolmates, teachers, romantic partners, and other members of the community throughout the whole life cycle [1, 6, 12–15]. Promotion of bonding means to develop and foster a strong affective relationship with and a commitment to parents or caregivers, positive peers, best friends, teachers in the school, romantic partners, and mature and caring adults in the community [1–3].

## 3. Theories of Bonding

There are numerous ecological, interpersonal, and biosocial theories of bonding which provide analyses from the macro-, mezzo-, and the microlevels on individual development within numerous significant contexts. These three perspectives provide different explanations of adolescent development [16]. Although perhaps seeming competitive, these perspectives are neither mutually exclusive nor incompatible. A critical examination illustrating their complementary nature is crucial for a comprehensive understanding. Each perspective provides a partial explanation for bonding and has important preventive, treatment, and policy implications. Indeed, a comprehensive explanation of bonding will likely involve a combination of factors. Initially, we review the three main conceptual perspectives guiding current research on bonding in adolescents.

### 3.1. Ecological Perspectives

The ecological perspective emphasizes complex interactions between persons and their environments. It emphasizes that human development is influenced by interactions between the biological and psychological factors of the adolescent and his/her environment, including the family, peers, the school, and the community [17]. It asserts that proximal settings are connected to each other and are contained in broader institutional and community contexts (macrosystems) which are located in particular historical, geographical, cultural, social, economic, and political contexts. This perspective, according to Collins and Steinberg [16], “integrates consideration of individual changes and the contexts (including relational contexts) that facilitate or interfere with those changes” (page 1011). Bronfenbrenner [18] defined the ecological approach to human behavior as the “scientific study of the progressive, mutual accommodation, throughout the life course between an active, growing human being and his or her environment” (page 188) and highlighted the importance of having several support systems and the interrelationship among these systems, particularly for an individual who needs to seek help [19]. Bonding in different systems provides support in different domains, for example, parents provide more advice regarding occupation and future goals to adolescents while peers are more influential in recreational activities or decisions in daily life [14, 20]. At least two child and adolescent development theories provide a central role for bonding under the ecological perspective: social control theory and the social development model.

#### 3.1.1. Social Control Theory

Hirschi asserted that social bonds explain why adolescents often do not seek immediate gratification in the easiest way possible [21]. As conceived by Hirschi, social bonds promoting socialization and conformity include involvement, attachment, commitment, and belief. He claimed that the stronger these four bonds, the less likely an adolescent would become delinquent. The first bond is *involvement in the socialization agent*. This addresses a preoccupation with activities which stress the conventional interests of society. The second bond is *attachment or affective relationships*, which refers to one's interest in others, including attachment to parents, to school, and to peers. Acceptance of social norms and the development of a social conscience depend on attachment. The third bond is *investment or commitment to the socialization agent* which involves time, energy, and effort placed on conventional lines of action. In other words, the support of and participating in social activities ties an individual to the moral and ethical code of society. The final bond is *belief in the values of the socialization agent*. It deals with the adolescent's assent to society's value system—which entails respect for laws and the people and institutions which enforce such laws. These social bonds, once strongly established, exert an informal control on adolescents' behavior, inhibiting deviant behavior in particular.

 Implications of the social control theory for positive youth development in general and for the promotion of bonding in particular are (1) attachment to parents as a result of the depth and quality of the parent-child interaction acts as a primary deterrent to engaging in delinquency; (2) attachment to school depends on how one appreciates the institution and how he/she is received by fellow peers and teachers; (3) attachment to parents and school overshadows the bond formed with one's peers.

#### 3.1.2. Social Development Model

The social development model of Catalano and Hawkins [22] integrates perspectives from social control theory, social learning theory, and differential association theory that together also suggest a central role for bonding. According to this model, children and adolescents must learn patterns of behavior from their social environment through four processes: (1) perceived opportunities for involvement in activities and interactions with others; (2) actual involvement; (3) acquiring skills for involvement and interaction; (4) perceived rewards from involvement and interaction [7]. Similar to social control theory, it hypothesizes the predominant behaviors, norms, and values held by those individuals or institutions will affect the behavior of the individual and influence them to become either prosocial or antisocial. Empirical support for the effects of bonding on both positive and problem behaviors has also been found [4, 8, 21, 23].

### 3.2. Interpersonal Perspectives

Three formulations (interdependence models, attachment perspectives, and social-psychological perspectives) typify the various interpersonal perspectives that emphasize how adolescents' experiences in social relationships change and subsequently contribute to individual development. According to Collins and Steinberg [16], these three formulations “differ primarily in the degree to which changes in dyadic relationships are attributed to individual maturation or to constraints and demands from larger contexts (e.g., schools). All three, however, assign a significant developmental role to the interactions that occur within dyads and social groups” (page 1011).

#### 3.2.1. Interdependence Models

In contrast to the independent model which assumes that the “ideal” and “healthy” individual is a self-sufficient and independent person, interdependence models examine how joint patterns of actions, cognitions, and both positive and negative emotional qualities in a close relationship between two individuals influence each other's thoughts, emotions, and actions [24, 25]. According to this perspective, adolescents' bonding to friends and lovers becomes more influential on the basis of shared interests, commitments, and intimacy; however, bonding to parents still remains significant although parent-child expectations need to be adjusted on both sides to maintain satisfactory interdependence. Conflicts in both types of bonding often result in expectation adjustments that lead to restoring equilibrium [26, 27]. The major implication for interdependence models is that adolescents develop as a result of balancing continuity and change as well as independence and interdependence.

#### 3.2.2. Attachment Perspectives

While the interdependence perspectives focus on behaviors, attachment perspectives emphasize the strong emotional ties between parents and adolescents and describe how parent-child bonding serves as an internal model that affects the child's future relationship with others. Bowlby [28] asserted that “attachment behavior is held to characterize human beings from the cradle to the grave” (page 129). His meaning is that the effect of bonding is life-long and will transfer among different kinds of relationships [2, 29, 30]. Ainsworth [6] listed six types of affectional bonds throughout the life span: (1) mother to infant; (2) father to child; (3) friendship; (4) companionship; (5) bonds between siblings and other kin; (6) bonding with a romantic partner. Bowlby argued that the fundamental need to establish contact and connection has adaptive roots in biological survival, and his attachment theory emerged as a major paradigm for empirical study of the mother-child relationship [28]. Even attachment with significant others during infancy and childhood has important consequences for a child's later development, Bowlby [30] believed that the attachment from adolescents to their parents still remains strong, although they may also have developed important bondings with peers and significant others.

 Thus, parent-adolescent bonding is both essential and significant during adolescence [31]. Many studies have suggested that having a secure relationship with their parents would have positive influences on adolescents' subsequent adjustment and healthy development [31, 32].

 A key implication of attachment perspectives is that when children grow up in a social environment that provides sensitive and responsive interactions with strong emotional ties, this facilitates well-adjusted adaptation during the transitions of adolescence.

#### 3.2.3. Social-Psychological Perspectives

During the transition from childhood to adulthood, multiple adaptations are required to respond to age-related changes in expectations, tasks, and settings [33]. Three major sources of impact on interpersonal relationships have been identified [16]. The first is the increase in anxiety arising from adapting to the multiple changes of early adolescence. The second is parent-child conflict as a result of the changes in the adolescent in adapting to the outside world [34]. The third is the pressure to reduce dependence on the family when adapting to extrafamilial contexts [35]. Such pressures affect adolescents' self-esteem, perceived independence, valuing of independence, methods of control, and overt behaviors [26]. Subsequently, these changes will affect the quality of bonding.

 A key implication of the social-psychological viewpoint is that adolescents will go through an increase and then a decrease in relationship difficulties from early to late adolescence [36] and the course of their development may encounter more accidental influences than implied by other theories.

### 3.3. Biosocial Perspectives

Two formulations provide a central role for bonding from the biosocial perspective. *Evolutionary perspectives* explain transformations in bonding during adolescence, mainly from the perspective of evolutionary psychology which views bonding as a strategic behavior that increases productive fitness [37, 38]. *Behavioral genetics* take into accounts research findings on the joint influence of biology and environment and uses statistical methods to differentiate the influences of bonding from (1) genetic influences; (2) shared environmental influences, such as siblings and socioeconomic status; (3) nonshared environmental influences, such as differential parental treatment and school experiences [39, 40]. These perspectives demonstrate how adolescents' intraindividual biological processes can help explain differences in interpersonal experiences. Although most behaviors are influenced by both nature and nurture, the nonshared elements generally have a stronger influence than shared environment [41]. It has also been found that shared family environment explains only 5% to 10% of the variance in behaviors and attitudes [42].

### 3.4. Remarks

Any perspective on adolescent bonding is bound to have limitations because it represents just one way of examining reality. Ecological, interpersonal, and biosocial theories address different dimensions of development. Although different theories of bonding have found some limited support in practical application and empirical testing, they remain falsifiable in different psychological, social, cultural, legal, and economic contexts. Furthermore, intervention programs based only on a single theory usually fail to adequately accommodate the multiple, qualitatively different layers of context. Nevertheless, a multidimensional perspective on adolescents' bonding provides a comprehensive understanding although it is practically very difficult to specify it to the extent necessary to guide empirical research.

## 4. Bonding and Positive Adolescent Development Outcomes

While infants need a secure attachment with caregivers, adolescents also need a sense of security and the encouragement to explore as they develop towards independent and autonomous individuals [43] through building both social and nonsocial bonds, including culturally based beliefs, traditions, values, and institutions. These bonds formed later in life provide benefits similar to infant attachment and parental bonding, such as a sense of security, comfort in stressful situations, guidance and support in decision making, physiological regulation, as well as long-term mental and physical health benefits [2].

 Certain specific bonds may be more likely to develop at different stages, including peer bonding in latency, commitment to social/cultural values during adolescence, and bonding to romantic partners after puberty. At the adolescent stage, peers, cultural belief systems, traditions, values, and associated institutions are important socialization agents where youngsters turn for emotional support and conformity [2, 6, 13, 44]. Owing to the changing environment and the development of adolescents' social and cognitive skills, bonding with peers and teachers may begin to substitute for bonding with parents [26, 29]. On the other hand, adolescents still look for full support from their parents [27, 29]. Therefore, simultaneously supporting adolescents to build and maintain bonds with parents, friends, teachers, and mature adults in the community can facilitate their whole-person development.

### 4.1. Bonding with Parents

Theorists and researchers have indicated that the types of parent-child bonding (the first bonding) will affect one's development of interpersonal relationships as one grows [2, 45–47]. For instance, children having a secure attachment with parents are more likely to become healthy and functional adults [3, 47]. They will grow up with high self-esteem, self-confidence, self-understanding, self-regulation, social competence, and better skills in problem solving and in building quality friendships [2, 3, 47]. Although adolescents probably will change their attachment object from their parents to peers or teachers, they still want full support from their parents [29]. Thus, it is vital to promote their bonding with parents.

 The importance of the attachment relationship with parents or caregivers is well documented. Based on a comprehensive study of 90,000 American teenagers, Blum and Rinehart [48] concluded that “across all the health outcomes examined, the results points to the importance of family and the home environment for protecting adolescents from harm. What emerges most consistently as protective is the teenager's feeling of connectedness with parents and family” (page 31).

 In addition, research studies have shown that family relationships during the adolescent period have important follow-on effects in a number of domains, such as autonomy and later independence of the individual [49], adolescent personality [50], individual pathology [51], and problem behavior [52]. While parenting styles influence the social and emotional development of adolescents, parents transmit their values and morals to their children which include beliefs about acceptable behaviors. Finally, parents are a vital source of information on a range of topics [53]. Litovsky and Dusek [54] also pointed out that adolescents who view their parents as warm, accepting, and providing them autonomy feel better about themselves and have more opportunity to practice social skills than those adolescents who perceive their parents as controlling, cold, and rejecting. Studies have also found that college students who are securely attached to their parents show better psychological and social adjustment and academic performance during their transition to college than students who are insecurely attached [55, 56].

 In short, positive outcomes from secure bonding to parents include a stronger sense of identity, higher self-esteem, greater social competence, better emotional adjustment, and fewer behavioral problems than less securely attached peers [57].

 On the other hand, maladaptive bonding with parents may lead to negative consequences such as parent-child conflicts when the parent-child dyad cannot strike a balance between the adolescents' need for autonomy and the parents' perception of connectedness. It is because when adolescents express their own individuality, parents with maladaptive bonding may take it as a sign of rejections and weakening relationships [58]. Although conflict management processes vary across parent-child dyads, the significance of a disagreement depends on the perceived quality of the relationship. Hauser and his colleagues [59] suggested that feelings of positive bonding promote the use of alternatives in a nonthreatening way whereas disagreement may be interpreted as a hostile attack that justifies an antagonistic response in maladaptive bonding. Furthermore, it was found that adolescents whose parents use a great deal of enabling and little psychological and behavioral control show higher level of individuality and score higher on measures of psychological competence and ego development [59, 60].

### 4.2. Bonding with Peers

Peers provide companionship, stimulation, physical support, ego support, and intimacy [61, 62]. Adolescents having friendships with more positive features reported having greater involvement in school and higher self-perceived social acceptance [12]. Moreover, adolescents with a positive bonding with close peers were more likely to develop a closer friendship with other peers [12, 63]. Therefore, developing adolescents' bonding with positive peers is important to their psychological health development and their social life.

 However, there are also extensive studies reporting the negative aspects of “peer pressure” or peer influence on adolescents [64, 65], including being excluded or rejected by peers, victimized by bullies, dumped by romantic partners, and detested by enemies. Social learning theory and primary socialization theory, as summarized by Kobus [66], suggest that peer relationships can be negative if the adolescent learns and acquires negative behavior from their friends, for example, smoking and substance abuse. Some researchers have taken a more optimistic view of peer pressure and suggest that the negative magnitude of friends' influence may be overestimated. For example, Bauman and Ennett [67] concluded the peer influence on drug use is exaggerated. Instead, attachment to a peer group can help adolescents avoid the problem of alienation [68], and interventions have successfully used the positive aspects of peer relationships to benefit delinquent youths [69]. Crosnoe and Needham [70] also found that adolescents with high-achieving friends in schools and high levels of bonding had the least behavioral problems. They add “in the adolescence stage, friendships enable adolescents to meet a key developmental task establishing their own lives independent from their families by helping them develop identities, test conventional boundaries, and gain autonomy” (page 265). In conclusion, peers may push each other towards risks and delinquent behaviors or help each other develop positively [71, 72].

### 4.3. Bonding with Teachers

Students in a supportive school environment (in which teachers are helpful but firm and maintain high, clearly defined standards for academic work and behavior) develop stronger bonds to teachers and the school and show higher achievement motivation. This bonding, in turn, helps adolescents have fewer problems, higher attendance, fewer incidents of delinquency, more supportive friendships, and higher academic performance [73, 74]. Another study by Howes and Aikins [75] found that a teacher who serves as an alternative attachment figure provides a secure base for new thoughts, promotes self-regulation, and leads to better friendship quality for adolescents. Moreover, Catalano and colleagues [7] provide empirical support for the theoretical propositions on the influence of school bonding, demonstrating the effectiveness of interventions to improve school connectedness and reduce a variety of health and safety problems, promote positive behaviors, and the attainment of academic success for children and adolescents. They concluded “school bonding appears to promote healthy development and to prevent problem behaviors” (page 252).

 Adolescent-teacher relationships are critical for the healthy development of the adolescent. Studies have shown that exposure to positive classroom climates and sensitive teachers are related to adolescents' greater self-regulation [76] and greater teacher-rated social competence [77]. Teachers exert influences on both prosocial and antisocial behaviors of adolescents; thus, teachers play a crucial role on the positive development of the adolescent [78].

 Blum and Rinehart [48] concluded that “school policies, classroom sizes, and teacher training appear unrelated to the emotional health and behaviors of students. Instead, what matters is the students' sense of connection to the school they attend: if students feel they are a part of the school, are treated fairly by teachers, and feel close to people at school, they have better emotional health and lower levels of involvement in risky behavior” (page 32).

### 4.4. Bonding with Romantic Partners

Carter and colleagues [2] concluded that emotionally close relationships developed during later childhood, adolescence, and adulthood are generally more mutual. These include “dyadic bonds” (individual to individual bonds), such as “love between parents and their older/adult children,” “sibling bonds,” “friendships in childhood and adulthood,” “bonds between sexual partners,” and “love between other biological relatives” (page 387).

 Psychologists suggest that a romantic relationship may emerge as an individual grows through adolescence [29]. Early romantic experiences play a crucial role in the development of the self and the ability to build up and maintain intimate relationships with significant others in the future. Interaction and relationships in the adolescent period with the opposite sex are believed to influence future romantic involvements and marriage in adulthood [79–81].

 According to Brown [82], adolescent romantic relationships develop through four stages: (1) initiation; (2) status; (3) affection; (4) bonding. The focus of the first stage is on testing oneself as a person capable of relating to the opposite sex in a romantic way. During the second stage, peer approval is needed in order to maintain or raise one's status in the large peer group. In the third stage, romantic relationships become more personal and caring. In the bonding phase, together with a long-term commitment, the emotional intimacy achieved helps create a lasting attachment.

 Sternberg [83] proposed that love consists of three basic ingredients: intimacy, passion, and decision/commitment. The intimacy component refers to feelings that promote closeness, bonding, and connectedness. The passion component refers to sources of arousal that promote the experience of passion, such as sexual needs, needs for self-esteem, affiliation, and submission. While the decision/commitment component refers to the decision that one is in love with another and the commitment to maintain that love. In short, adolescent dating serves multiple purposes, including recreation, autonomy seeking, status seeking, sexual experimentation, social skills development, and courtship [84].

 Studies suggest that dating at an early age may have more negative than positive outcomes. This may be either because troubled adolescents start dating early or because they get hurt in dating or they become involved in teenage problem behavior [85, 86]. However, both secure bonding with parents and same-gender peers can protect young adolescents from the negative effects of early dating [87, 88].

 In general, dating typically has more positive than negative developmental outcomes. Involvement and commitment in a steady relationship promote self-esteem and better overall adjustment [85]. Securely attached college students who were able to keep a close and caring bond with their parents were found to be able to form new relationships with romantic partners. On the contrary, resistantly attached college students experienced more difficulties entering into romantic relationships [89].

### 4.5. Remarks on the Gender Issue of Bonding

Research on the development of same-sex and other-sex friendships has indicated that intimacy is more important and emerges earlier for girls than boys [90]. It has also noted that females focus more on self-disclosure and mutual help [91].

 Among older adolescents, girls initially emphasize the interpersonal aspects of romantic relationships such as commitment and self-disclosure, whereas boys focus more on their partners' physical attractiveness and sexual relations [92]. Whereas boys show an interest in girls in a sexual way, girls are more interested in boys in a romantic way [93].

## 5. Promotion of Adolescent Bonding

Secure bonding established in an adolescent's life with good friends, caring family members, mature adults in the school and the community will produce positive results in a number of ways.

**Promotion of Physical and Psychological Safety**

Bonding grows from safe and health-promoting peer, family, school, and community environments and facilities with practices that increase safe and meaningful parent-child and peer group interactions as well as a decrease in unsafe or confrontational parent-child and peer interactions. Schools and community facilities have to be safe and free from gangs and illegal or immoral activities.

**Establishment of Appropriate Structure That Promotes Bonding**

Adolescents develop and maintain their bonding in a predictable and dependable environment with appropriate limits, clear and consistent rules and expectations, proper age-appropriate internal and external monitoring and control, suitable balance between firmness and flexibility, and clear boundaries.

**Cultivation of Supportive and Intimate Relationships**

Open and frequent communication with respect, feelings of warmth, caring and closeness, availability of supportive guidance, and responsiveness from family members, friends, and mature adults in social systems facilitate bonding and promote healthy intimate relationships.

**Creating Opportunities to Belong**

In bonding with peers and others in the community, opportunities for meaningful social inclusion (regardless of one's gender, ethnicity, sexual orientation, or disability status) are necessary conditions, while opportunities for sociocultural identity formation as well as support for cultural and bicultural competence should also be made available.

**Promotion Positive Social Norms**

Adolescents need clear rules and behavioral expectations derived from prosocial values and morals. Prosocial norms can be reinforced through bonding to important socializing units, that is, the family, school, peers, and community.

**Support for Efficacy**

Attachment to peers with undesirable behavior yields problem behaviors and negative consequences. Positive youth development programs that promote self-efficacy will help adolescents counteract these negative influences. Youth-based empowerment practices or programs that support autonomy, making a real difference in one's community and being taken seriously, are major strategies. Practices that include enabling, responsibility granting, involvement in meaningful challenges, and focus on improvement rather than on current relative performance level can support adolescents to build their self-efficacy.

**Provision of Opportunities for Skill Building**

Parents, teachers, mature adults in the neighborhood or community who provide adolescents with opportunities to learn physical, intellectual, psychological, emotional, and social skills; reflect on intentional learning experiences; learn cultural literacy, media literacy, communication skills, good mental habits, and employment-related skills help build bonding with adolescents and develop their social and cultural capital.

Moreover, good communication and relationships with others including trust, empathy, active listening, expression of feelings, mutual help, intimacy, self-disclosure, acceptance, mutual affection, emotional support, emotional stability, and extroversion all contribute to bonding [12, 13, 46, 63, 91, 94]. Hence, it is necessary to teach adolescents to acquire these positive features in order to bond with significant others in different systems.

## 6. Cultural Issues

One of the most significant cultural heritages of a Chinese society is that the core values of Confucianism place a great emphasis on building harmonious interpersonal relationships. Shek and Chan [95] reported that Chinese parents consider bonding especially the quality of the parent-child relationship and the obedience of the child as the most important attributes of an “ideal child.” Chen et al. [96] suggested that Chinese often form small well-defined “cliques” in contrast to Canadians. Chao and Tseng [97] reported that Chinese mothers and fathers play very different roles in their parent-child relationships. Ho [98] suggested that the difference between paternal and maternal parenting styles is well reflected in a traditional saying “strict father, kind mother.” The role of the mother is to provide a secure and warm home environment and to develop a close and emotional relationship with children. On the other hand, the role of father is to provide economic support and moral instruction, rather than a (adjective) emotional relationship. A “traditional” father would love his child, but he seldom expresses his love verbally. However, a recent study by Shek [99] found that the situation has been reversing—the notion has changed to “strict mother, kind father.” Results also showed that the quality of parental control has declined and parent-child relational qualities have become poorer in early adolescent years in the contemporary Chinese culture of Hong Kong.

### 6.1. Similarity or Difference

The development of attachment would seem to be a necessary, universal biological requirement to be found in all cultures under normal circumstances as a species-specific consequence of our phylogenetic heritage. However, even if the attachment system is biologically based and universal, this in no way contradicts the principal of cultural mediation. The biological system of attachment is interwoven with cultural practices [10]. Research has initially suggested significant variation in the proportion of infants showing each pattern of attachment-related behavior. For example, when at the age of 11 to 14 months communally reared Israeli children were placed in the Strange Situation, many became very upset; half were classified as anxious resistant, and only 37% appeared to be securely attached [100]. Researchers suspect that cultural differences in the opportunities for sensitive caregiving accounted for differences in attachment quality. Moreover, attachment behaviors will differ in distinct cultures and in different epochs depending on differences in customs of child care, family or social structure, devastating or benign living conditions, and similar environmental circumstances [101].

 On the other hand, a comparison of behaviors of mother-child pairs observed in their homes in the United States and Uganda by Ainsworth [102] found that children in both cultural groups exhibited similar patterns of attachment-related behavior although the Ugandan children seemed to express these behavior patterns more readily and intensely than did the American children. Most studies show that, across a variety of cultural settings, about two-thirds of the attachments to either parent are rated secure [11].

 Across many cultures, secure attachments (Type B) are the most common. However, in places like Israel, Japan, Indonesia, and China, insecure-ambivalent attachments (Type C) appear more often than in other places. This most likely results from cultural differences in parenting styles [103]. These could mean that parents in some countries are more or less sensitive than American parents, but this ethnocentric interpretation seems incorrect. The strange situation would not be psychologically similar for these babies and American babies (the psychological meaning of the procedure for infants from each culture may differ).

 A low percentage of securely attached babies have also been observed among northern German children. Researchers in one study found that 49% of the 1-year-olds tested were anxious avoidant and only 35% were securely attached [104]. The researchers rejected the possibility that a large proportion of northern German parents were insensitive or indifferent to their children. They suggested that northern German parents were adhering to a cultural value that calls for the maintenance of a relatively large interpersonal distance and to a cultural belief that babies should be weaned from parental bodily contact as soon as they become mobile. In Japan, Miyake and his colleagues found a large proportion of anxious-resistant infants among traditional Japanese families and no anxious-avoidant infants at all [105, 106]. They explained this pattern by pointing out that traditional Japanese mothers rarely leave their children in the care of anyone else, and they behave toward them in ways that foster a strong sense of dependence. Consequently, the experience of being left alone with a stranger is unusual and upsetting to these children. In general, Western industrialized cultures tend to be viewed as individualistic with an emphasis on self-actualization, whereas Eastern cultures and those that are less industrialized tend to be viewed as collectivistic with an emphasis on interdependence [107, 108].

 Chinese are comparatively more concerned with interpersonal harmony and are more likely to have close relationships within the family. However, the individualism-collectivism dimension does not provide a general theoretical model for distinguishing among cultural groups [109]. The evidence of cultural variation has been brought into question and balanced by evidence that there is a general tendency in all societies for children to become attached to their caregivers. In an influential review of research on attachment in different cultures conducted by van IJzendoornm and Sagi reported that although the proportion of children displaying one or another pattern of attachment behaviors may vary in a small number of cases, the overall pattern of results is remarkably consistent with Ainsworth's initial findings [11]. The global distribution was found to be 21% Type A (anxious avoidant), 65% Type B (securely attached), and 14% Type C (anxious-resistant), with greater variation within countries than between them. When Behrens et al. [110] replicated Miyake's research with older Japanese children, they found a distribution of A, B, C categories similar to worldwide norms.

### 6.2. Concluding Remarks

Assessing culture divergence in social attachment is nearly impossible. As described by Gjerde [111], culture is a rapidly moving target, continually changing in a context of economic, political, and historical forces. Secondly, individuals within a given cultural group are heterogeneous, and there are often greater differences within cultures than across cultures. Furthermore, cultural effects are confounded with other cultural variables, such as social class, economic conditions, and geographic locations. Therefore, it is expected that wide variation can exist within a given culture.

 Longitudinal studies have documented both continuity in attachment relationships quality from infancy to early adulthood and also discontinuity, with the latter being meaningfully related to changes in the lives of individuals and their family environments [112–114]. Specifically, evidence supports the notion that negative life events (e.g., loss of a parent, parental divorce, life-threatening illness of parent or child, and parental psychiatric disorder) could bear on the caregiver's availability and responsiveness, which impacts child-parent interactions and in turn affects children's security [115]. The patterning of past interactions, present exchanges, and the ecology of the dyad all seem at play in helping account for individual differences in child-parent attachment relationships [116].

## 7. Future Research

In future research, it is important to go beyond the view of bonding as solely a stress-reducing system and study the operations and functions in terms of emotional attachment and commitment during ordinary everyday circumstances. Attachment and commitment are built in the context of regular exchanges that include positive nonemergency situations that both children and parents enjoy [116]. As noted earlier, research points to important interrelations among adolescents' relationships with friends. The nature and processes of this developmentally significant network of relationships promises to become an increasingly prominent focus of future research. Moreover, further research on the gender identity development of adolescents and young adults is needed to guide practice in high school and college contexts.
